# Moraxella Bacteremia in Cancer Patients

**DOI:** 10.7759/cureus.15316

**Published:** 2021-05-29

**Authors:** Shamra Zaman, John Greene

**Affiliations:** 1 Medicine, University of South Florida, Tampa, USA; 2 Internal Medicine, Moffitt Cancer Center, Tampa, USA

**Keywords:** moraxella, myeloma, respiratory tract, pneumonia, immunocompromised patient

## Abstract

*Moraxella* is a gram-negative bacterium part of the *Moraxellaceae* family. It is a pathogen that is commonly found in the upper respiratory tract of humans. It is a rare cause of community-acquired pneumonia and can be found in immunocompromised individuals, especially those with impaired humoral immunity such as hypogammaglobulinemia and those with lung diseases.

We present three cases of *Moraxella *infections at the Moffitt Cancer Center between the years 2011 and 2017. We performed a literature review of *Moraxella* bacteremia in cancer patients and included three patients, two with a history of multiple myeloma and one undergoing radiation therapy for non-small cell lung carcinoma. None of the patients died as a result of the infection. *Moraxella* infections can result in a range of severity with increasing resistance to antibiotic therapy.

## Introduction

*Moraxella* is a gram-negative bacterium that has a *coccobacillus *shape [1]. Originally considered normal flora in the human respiratory system, it can cause respiratory tract infections [2]. It primarily affects adults with prior chronic lung disease and the immunosuppressed. The most common immunodeficiency is hypogammaglobulinemia, which is found in patients with multiple myeloma and chronic lymphocytic leukemia (CLL). Invasive infections include meningitis, pneumonia, and endocarditis [3,4].

We present the cases of three cancer patients with *Moraxella* infections that illustrate the most common risk factors that predispose to this infection.

## Case presentation

Case 1

A 62-year-old Hispanic male presented with a fever and cough. He has a significant history of multiple myeloma and underwent an autologous stem cell transplant in 2007, four years prior to consultation. Prior infections included herpes zoster on the back and genital herpes. Eventually, the relapse of myeloma required chemotherapy complicated by neutropenia. His medical history consisted of hypertension, prostate hypertrophy, folliculitis on the scalp, hyperlipidemia, hemorrhoids, and kyphoplasties of the spine. He was hospitalized with pneumonia requiring a short hospitalization early in 2011. He had reported having one cat as a pet with no bites or scratches.

**Figure 1:**
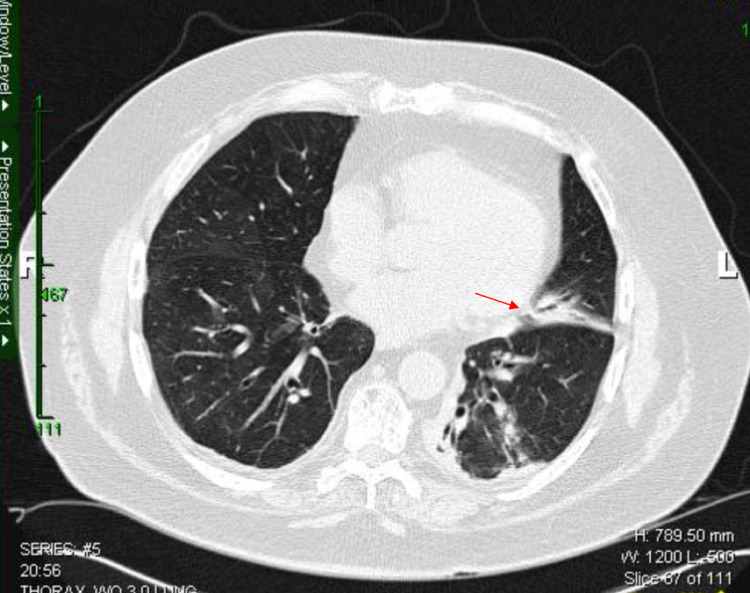
CT scan of the thorax showing the focal area of consolidation (red arrow)

Upon physical examination, he presented with fever with a temperature of 100.9°F. The white blood cell count was 0.78 cells/uL with a neutrophil count was 460 cells/uL, immunoglobulin (Ig) G was 498 mg/dL, and IgA and IgM were undetectable. CT scan revealed maxillary polyps and maxillary sinusitis. CT also presented areas of consolidation (Figure 1). One of two peripheral blood cultures grew gram-negative *diplococci* bacteria subsequently identified as *Moraxella*, and the sputum culture grew *Candida*. The patient was initially treated with cefepime, tobramycin, and acyclovir. After improvement, he was discharged three days later and completed a seven-day course of oral levofloxacin.

Case 2

A 60-year-old African American female was diagnosed with lambda multiple myeloma and presented with renal insufficiency. Several months prior to admission, she was treated for sepsis and a pathological fracture of the right proximal femur and placed on lenalidomide. After beginning Revlimid®, she developed Steven-Johnson syndrome resulting in intubation in the intensive care unit. After recovery, she continued chemotherapy and became dependent on hemodialysis. She was again admitted for sepsis, and a new distal femur pathologic fracture was discovered the following year. Several months later, she was admitted to Moffitt Cancer Center, Tampa, Florida, from a rehabilitation facility for failure to thrive.

**Figure 2:**
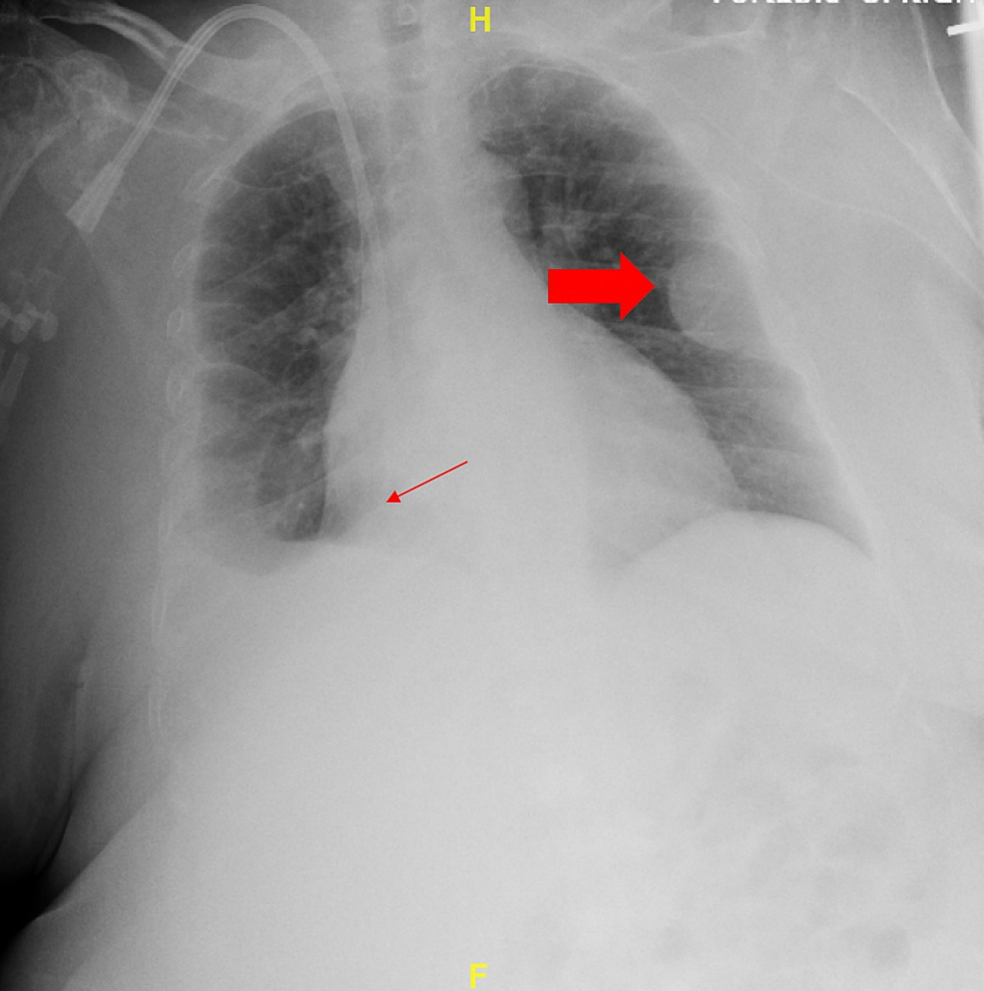
Image showing X-ray of the chest Right-sided pleural effusion (thin arrow) and left-sided pleural-based mass (thick arrow) can be seen.

The white blood cell count was 3.9 K cells/uL, with a lymphocyte count at 1.5 cells/uL. IgA and IgM were undetectable. Chest X-ray, posterior-anterior and lateral, showed a moderate right pleural effusion and a left lateral sixth rib pleural-based mass (Figure 2). The bone survey showed multiple lucencies throughout the skeleton. CT scan of the chest demonstrated multiple osteolytic bone lesions, right humeral head fracture, and a moderate right pleural effusion with secondary right lung atelectasis. *Moraxella catarrhalis *bacteremia was found on two blood cultures.

She was treated with seven days of ceftriaxone. She recovered from this infection but died the following year from myeloma progression and renal failure.

Case 3

A 77-year-old female patient with a history of coronary artery disease, chronic obstructive pulmonary disease, and gastroesophageal reflux disease was diagnosed with non-small cell lung carcinoma and treated with radiation therapy. She had completed three of the five radiation treatments when she presented with abdominal pain and fever of 101.9°F that required hospitalization.

The white blood cell count was 13.45 cells K/uL. Physical findings included injection site cellulitis from intravenous Ig infusion. Blood cultures grew gram-negative rods identified as *Moraxella osloensis*. She was treated with intravenous cefepime. Five days after hospitalization, she improved and was discharged on oral ciprofloxacin for 14 days.

## Discussion

Patients undergoing treatment for lung cancer, myeloma, and CLL are highly vulnerable to respiratory infections. *Moraxella* bacteremia may present with minimal respiratory symptoms and radiographic findings [5-7]. The most common genus includes *Moraxella catarrhalis*.

In this study, we illustrate the most common risk factors for *Moraxella *infections. All cases presented were immunosuppressed with a history of lung cancer or multiple myeloma that resulted in hypogammaglobulinemia. One of the patients had an invasive infection with *Moraxella osloensis*, a rare *Moraxella* species.

*Moraxella* is a pathogen that is susceptible to most beta-lactamase stable antibiotics and fluoroquinolones [8-11]. The choice of antimicrobial agents depends on the age of the patient, comorbid conditions, and desired antimicrobial coverage [12]. All cases responded to beta-lactamase stable antibiotics and fluoroquinolones such as levofloxacin and ciprofloxacin. Hwang found 90% of *Moraxella catarrhalis* strains to be resistant to penicillin and amoxicillin due to the production of β-lactamases [13]. Another study in 2002 by Schmitz et al. found that 98% of *Moraxella *isolates produce beta-lactamases [14].

## Conclusions

*Moraxella* infections develop in older adults who have structural lung diseases such as emphysema, chronic obstructive lung disease, and lung cancer. In addition, patients with humoral immunodeficiency due to hypogammaglobulinemia from multiple myeloma and CLL are especially vulnerable to respiratory infections that may result in bacteremia. *Moraxella* can cause invasive infection in some patients with minimal respiratory symptoms. Because of the frequent production of beta-lactamase, therapy with a beta-lactamase stable antibiotic or fluoroquinolone is preferred. The prognosis from infection is excellent, but the underlying malignancy and other comorbid conditions ultimately cause the patient’s demise.
